# Prevalence and Correlates of Adynamic Bone Disease in Patients with Kidney Failure in Singapore

**DOI:** 10.3390/jcm15103758

**Published:** 2026-05-14

**Authors:** Siew Kit Shuit, Erin Yan Qing Wee, Yuan Kai Teh, Fangxia Chen, Regina Shaoying Lim, Manohar Bairy

**Affiliations:** 1 Department of Renal Medicine, Tan Tock Seng Hospital, 11 Jalan Tan Tock Seng, Singapore 608433, Singapore; siewkit.shuit@mohh.com.sg (S.K.S.); teh.yuan.kai@nhghealth.com.sg (Y.K.T.); fangxia.chen@mohh.com.sg (F.C.); regina.lim@nhghealth.com.sg (R.S.L.); 2 Lee Kong Chian School of Medicine, Nanyang Technological University, 11 Mandalay Road, Singapore 308232, Singapore; ewee007@e.ntu.edu.sg

**Keywords:** adynamic bone disease, parathyroid hormone, renal hyperparathyroidism

## Abstract

**Background/Objectives:** The spectrum of chronic kidney disease—mineral and bone disorder (CKD-MBD) is changing and adynamic bone disease (ABD) is now believed to constitute the majority of CKD-MBD in the developed world. However, its prevalence and risk factors are poorly described in the literature. Its diagnosis requires bone biopsy, but biochemical criteria including parathyroid hormone (PTH) levels show good correlation. The aim of this study is to understand the prevalence of ABD in our patients with kidney failure (KF) on hemodialysis (HD), identify the risk factors for its development, and in doing so enable early intervention to modify the risk factors specific to our population. **Methods:** This is a retrospective cross-sectional study. A total of 201 prevalent adult patients on maintenance HD for at least 3 months were recruited. Patients with previous parathyroidectomy were excluded. Relevant data including clinical and biochemical parameters, prescribed dialysate and medications, and clinical outcomes were collected. ABD was diagnosed if any intact PTH (iPTH) level during the study period was <15 pmol/L. **Results:** Of the 201 patients in the study (median age 64.5 years), 35 (17.4%) patients had ABD. In the multivariable logistic regression model, the adjusted odds ratio (OR) of ABD was higher with a higher mean adjusted serum calcium level, while the concurrent use of non-calcium-based binders was associated with lower odds of ABD. Activated vitamin D use was also associated with lower odds of ABD, likely reflecting past occurrence of ABD, prompting a pre-emptive discontinuation of activated vitamin D. Overall, 17% of patients had had fractures without significant association with ABD. The mean PTH level was in the target range (15–60 pmol/L) in 41% of the cohort. Cardiovascular complications were not significantly associated with ABD. **Conclusions:** Approximately one in every six HD patients in our care have ABD as diagnosed by the iPTH level. Targeting a lower serum calcium level and using non-calcium-based binders may reduce the occurrence of ABD and will need to be tested in prospective studies.

## 1. Introduction

The global burden of patients with kidney failure (KF) is steadily increasing [1,2,3], accompanied by changes in patient demographics, characterized by an ageing population [1] with longer survival [4] and a higher prevalence of comorbid conditions [5]. Chronic kidney disease—mineral and bone disorder (CKD-MBD) is a common complication of kidney failure. There has been a change observed in the spectrum of CKD-MBD in recent years, with an increasing prevalence of adynamic bone disease (ABD) reported in the dialysis population [6], likely due to advanced age [7], increased prevalence of diabetes mellitus (DM) [8,9], and more intensive treatment of secondary hyperparathyroidism with more vitamin D analogues or calcimimetics being used [10]. The reported prevalence of ABD in KF varies widely, and is estimated to be around 10–50% depending on the population studied and the diagnostic criteria used [11]. However, the literature on ABD and its determinants in the KF population is sparse.

The diagnosis of ABD remains challenging in clinical practice. The gold standard for diagnosis of ABD is from bone biopsy. However, biochemical markers such as intact parathyroid hormone (iPTH) and alkaline phosphatase (ALP) levels show good correlation with ABD diagnosed by bone biopsy. Data from metanalyses suggests that low iPTH levels are associated with an increased likelihood of low bone turnover, supporting its use as a pragmatic biochemical surrogate in the absence of bone histomorphometry [12]. An iPTH level of less than two times the upper limit of normal (ULN) has shown moderate diagnostic performance for identifying low bone turnover states, with reported sensitivity and specificity rates of 65 and 66%, respectively, to discriminate low from non-low turnover bone disease [13].

Risk factors of ABD can be broadly divided into over-suppression of the parathyroid glands and PTH resistance [14]. Apart from calcimimetics therapy and total parathyroidectomy, ABD can occur due to over-suppression of parathyroid glands due to increased calcium burden contributed by the use of calcium-based phosphate binders, higher dialysate calcium bath, and the use of vitamin D analogues or calcitriol [10,15]. Resistance to PTH actions on bone is more likely in patients with DM [10,16,17], and can also be seen in states of chronic inflammation and poor nutrition [14,18].

The clinical significance of ABD cannot be over-emphasized as it can be associated with a significant risk of vascular calcification [7], fractures [19] and all-cause mortality [20,21]. We aim to estimate the prevalence of ABD in KF and identify the risk factors associated with ABD, so that interventions can be focused on mitigating this risk.

## 2. Materials and Methods

### 2.1. Study Design

This is a retrospective analytical cross-sectional study. We identified a cohort of 1100 adult patients on maintenance HD on follow up with the Nephrology services in Tan Tock Seng Hospital, Singapore, between January and July 2024.

The required sample size was determined using Cochran’s formula, assuming a 15% prevalence of ABD, 5% precision level, loss due to missing data projected at about 5% and 5% confidence level, indicating a minimum of 196 participants. In total, 216 patients were selected using the simple random sampling method. Overall, 201 patients with complete data and meeting the study criteria were included in the study.

This study was approved by the Ethics Review Board (Domain Specific Review Board of National Healthcare Group, Singapore—Ref 2023/00526).

### 2.2. Inclusion and Exclusion Criteria

We included all patients with KF who had been on maintenance HD for at least 3 months. Patients who had a prior history of parathyroidectomy or incomplete biochemical data were excluded.

### 2.3. Data Collection

Data was obtained from electronic health records, including blood test results from dialysis centres that form part of the electronic health records uploaded during routine clinic consults at the hospital.

Data collected included patients’ demographics, including age, gender, etiology of KF, dialysis vintage, and history of DM; biochemical investigations, including serial serum levels of adjusted calcium, phosphate, iPTH, ALP, and albumin; treatment-related data including dialysate calcium, use of calcium-based and non-calcium-based phosphate binders, calcitriol or vitamin D analogue and calcimimetics; and complications like ischemic heart disease (IHD), stroke, peripheral vascular disease (PVD), fracture and mortality. Given the cross-sectional design, complications were assessed at the point of study inclusion based on patients’ clinical status at that time. Mortality events occurring prior to the conclusion of the study were recorded retrospectively.

For each patient, the two most recent measurements of iPTH and the three most recent values of serum calcium and phosphate were obtained. The most recent record of prescribed medications was collected to reflect current therapeutic management. In instances where iPTH levels were found to be below 15 pmol/L at any time point, the corresponding serum calcium and phosphate values, as well as the medication regimen, were aligned specifically with the time of iPTH nadir. This adjustment was made to accurately capture clinical context, acknowledging that iPTH over-suppression often prompts modifications in therapy. The reference range for iPTH in our centre is 0.8–6.8 pmol/L. However, as patients included in our study were from multiple community dialysis centres, iPTH measurements were performed using different assays across laboratories, and inter-assay variability may exist.

The prevalence of ABD was defined by an iPTH level of <15 pmol/L at any point. This threshold of 2 times the ULN was selected based on prior studies and meta-analysis demonstrating that an iPTH suppressed below this threshold had moderate diagnostic performance in distinguishing low and non-low bone turnover states [12,13]. Although serial iPTH levels were available and we acknowledge the importance of interpreting iPTH trends rather than relying on single measurements, we chose to use iPTH < 15 pmol/L to diagnose ABD instead of using a mean iPTH < 15 pmol/L, as using mean values may underdiagnose ABD and therefore underestimate the prevalence of ABD. The presence of a suppressed iPTH value was considered clinically significant, as such a value would usually prompt therapeutic modification in routine practice, such as the cessation of activated vitamin D analogues. Following such interventions, subsequent iPTH values are often higher, and averaging serial iPTH measurements may therefore attenuate the detection of clinically relevant episodes of PTH over-suppression and underestimate the risk of low bone turnover.

Missing data was imputed using mean substitution. There were two missing values for ALP, which were imputed using this method.

### 2.4. Statistical Analysis

Data analysis was conducted using Jamovi 2.6.44. Mean with standard deviation (SD) was used for continuous data if they were normally distributed or median with interquartile range (IQR) otherwise. We compared the demographic, laboratory and clinical parameters using a *t*-test or Mann–Whitney U test for continuous variables where appropriate. Chi-square test or Fisher’s exact test was used for categorical variables where appropriate. ABD was defined as iPTH < 15 pmol/L at any point in time. To identify the risk factors for ABD, a multivariable logistic regression model was constructed. Variables with a *p* value of <0.10 from univariable regression and important covariates based on past studies were entered simultaneously into the multivariable logistic regression model. The variance inflation factor (VIF) was calculated to check for multicollinearity in the multivariable regression model. Goodness-of-fit was assessed with McFadden’s pseudo-R^2^. Statistical significance was defined as *p* value < 0.05.

## 3. Results

**Figure 1:**
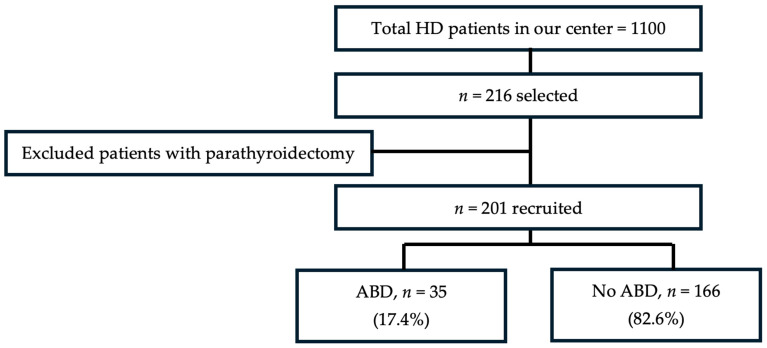
Patient flow chart and outcomes. Abbreviations: HD, hemodialysis; ABD, adynamic bone disease.

Among the 201 patients, 17.4% (*n* = 35) had ABD (Figure 1). The median age of the study cohort was 64.5 years; 61.2% (*n* = 123) were males and 61.7% (*n* = 124) had KF with DM closely matching the prevalence of DM in the local HD population. The average dialysis vintage was 41 months (IQR 18, 78) (Table 1). The median dose of calcium acetate was 1.33 g/day (IQR 0.0, 3.50) in the ABD group and 2.00 g/day (IQR 0.0, 3.67) in the patients without ABD. The median dose of vitamin D analogues was 0.00 microgram/week (IQR 0.0, 1.94) in the ABD group and 1.00 microgram/week (IQR 0.18, 3.00) in the patients without ABD.

In the univariable analysis, older age and lower serum ALP were both significantly associated with ABD. The use of non-calcium-based phosphate binders, calcitriol, and vitamin D analogues was also associated with lower odds of ABD (Table 2).

However, we incorporated other important variables like DM, dialysate calcium, and serum calcium into the multivariable model, as these factors, based on previous studies, were found to be significantly associated with the risk of ABD. As all patients on calcimimetics in the cohort (*n* = 22) did not meet the criteria for ABD, calcimimetics use was not included in the multivariable model due to lack of convergence. Variables such as gender, dialysis vintage, etiology of KF, serum phosphate, albumin, and use of calcium-based phosphate binders were not incorporated into the multivariable model as they were not significant in the univariate analysis.

In the multivariable logistic regression analysis, calcitriol or vitamin D analogue therapy and the use of non-calcium-based phosphate binders decreased the odds of having ABD, while elevated serum calcium levels increased the odds of having ABD (Table 3). Specifically, each 0.1 mmol/L increase in serum calcium was associated with 1.68-fold higher odds of ABD (OR = 1.68; 95% CI: 1.15–2.45; *p* = 0.007). Patients receiving calcitriol or vitamin D analogues had significantly lower odds of ABD (OR = 0.20; 95% CI: 0.08–0.50; *p* < 0.001). Non-calcium-based phosphate binder use was associated with a markedly reduced risk of ABD (OR = 0.05; 95% CI: 0.00–0.47; *p* = 0.008). The mean VIF of the multivariable logistic regression model was 1.16, suggesting the absence of significant multicollinearity. The model’s McFadden’s pseudo-R^2^ was 0.244, indicating an excellent goodness-of-fit.

We did not find that the presence of ABD was significantly associated with the risk of IHD, PVD, stroke, fracture and mortality (Table 4).

## 4. Discussion

The prevalence of ABD in our population was 17.4% using the criteria of serum iPTH level of <15 pmol/L. This varies from that reported in some previous studies, likely due to variations in diagnostic methodology and iPTH cutoff values used to define ABD [17,22]. While bone biopsy remains the gold standard for diagnosing ABD and has been associated with higher reported prevalence rates [17], its use is limited in routine clinical practice due to the limited availability of experienced operators and interpreters, as well as the procedure’s cost, invasiveness, and potential to cause pain [23,24]. Other studies have relied on biochemical markers, including iPTH levels [22], but have applied different threshold values for defining ABD, contributing to the difference in prevalence across studies. As the iPTH data were obtained from different laboratories due to the inclusion of patients from multiple community dialysis centres, variations in assay methods may have introduced inter-laboratory variability and potential measurement bias. Such variability in PTH measurement is well-recognized and may contribute to misclassification when absolute thresholds are applied.

We found that high serum calcium was associated with higher odds of developing ABD and the use of non-calcium-based phosphate binders was associated with lower odds of ABD. These findings are consistent with the current standard of care and the KDIGO 2017 CKD-MBD guidelines, which are to avoid hypercalcemia and restrict the dose of calcium-based binders. Interestingly, the use of calcitriol and vitamin D analogues was also associated with lower odds of ABD (OR = 0.20; 95% CI: 0.08–0.50; *p* < 0.001), which appears counterintuitive, as calcitriol and activated vitamin D analogues are traditionally considered risk factors for ABD due to their suppressive effects on iPTH and potential to reduce bone turnover [10,15]. One possible explanation is that this could reflect proactive discontinuation of vitamin D when iPTH levels were trending down. As such, patients with evolving ABD may be less likely to remain on calcitriol and activated vitamin D analogues at the time of assessment. The use of dialysate with 1.5 mmol/L of calcium and the use of calcium-based phosphate binders were not found to be significantly associated with the risk of ABD, which could also reflect a proactive approach of reducing calcium exposure when iPTH is trending down. However, we did not find that the risk of cardiovascular complications or fractures was higher in ABD. This could be explained by the small number of events and its being a cross-sectional study with no longitudinal follow up—the complications may have happened after the development of ABD.

This study has several strengths, including the inclusion of a sufficiently large sample size and minimal missing data, which enhance the reliability of the findings. To our knowledge, this is the first study in Singapore and in Asian populations to examine the correlation between ABD and its clinical and biochemical parameters, thereby increasing the relevance and generalizability of the findings to similar regional populations. Moreover, the identification of variables associated with the development of ABD offers valuable evidence that may inform targeted mitigation strategies. However, there are notable limitations. Being a single-centre study may limit the external validity of the results. The diagnosis of ABD was based on serum iPTH levels rather than definitive bone biopsy, which remains the gold standard. Interpretation of iPTH as a surrogate for bone turnover remains challenging due to its biological variability and imperfect correlation with bone histology. While KDIGO guidelines [25] recommend assessment of serial PTH trends, we deliberately did not use averaged iPTH values in our primary analysis. In clinical practice, the detection of a markedly suppressed iPTH level often prompts therapeutic modification, resulting in subsequent increases in iPTH. Averaging serial values may therefore obscure clinically meaningful episodes of PTH oversuppression, particularly in patients undergoing dynamic therapeutic modification. Our approach was intended to better capture such episodes and reflect real-world clinical decision-making, although we acknowledge that this may introduce some degree of misclassification. Future studies incorporating serial PTH trajectories may improve the phenotyping of low bone turnover states and reduce misclassification. In addition, the choice of iPTH threshold is also challenging. While we used a cutoff of <15 pmol/L to identify markedly suppressed PTH levels, prior studies have demonstrated only moderate diagnostic accuracy of PTH alone for bone turnover assessment. Nevertheless, suppressed PTH values remain clinically relevant and are associated with an increased likelihood of low bone turnover states. Bone-specific ALP and activated vitamin D levels were not measured, potentially limiting the assessment of bone turnover. The cross-sectional design also introduces temporal ambiguity, preventing causal inferences, and we are limited by the use of retrospective medical records. Finally, the study design does not allow for the evaluation of therapeutic interventions that may influence bone turnover dynamics.

## 5. Conclusions

Approximately one in every six HD patients in our care has ABD as defined by iPTH < 15 pmol/L. Targeting a lower serum calcium level and preferential use of non-calcium-based phosphate binders may reduce the occurrence of ABD. Prospective studies are needed to determine the optimal target serum calcium levels in patients with KF on HD, and to guide the clinical use of phosphate binders, activated vitamin D, calcimimetics, and dialysate calcium concentrations.

## Figures and Tables

**Table 1:** Baseline demographic and laboratory data—adynamic bone disease vs. no adynamic bone disease.

Variables	Overall (*n* = 201)	Adynamic Bone Disease (*n* = 35)	No Adynamic Bone Disease (*n* = 166)
Age, mean (SD), years	64.50 (12.50)	68.70 (12.50)	63.60 (12.40)
Gender, *n* (%)			
Male	123.00 (61.20)	21.00 (60.00)	102.00 (61.40)
Female	78.00 (38.80)	14.00 (40.00)	64.00 (38.60)
Dialysis vintage, median (IQR), months	54.00 (30, 80)	41.00 (18, 78)	56.00 (31, 90)
Etiology of kidney failure, *n* (%)			
Diabetes mellitus	124.00 (61.70)	25.00 (71.40)	99.00 (59.60)
Hypertension	34.00 (16.90)	5.00 (14.30)	29.00 (17.50)
Chronic glomerulonephritis	35.00 (17.40)	3.00 (8.60)	32.00 (19.30)
Others	8.00 (4.00)	2.00 (5.70)	6.00 (3.60)
Diabetes mellitus, *n* (%)			
Yes	144.00 (71.60)	29.00 (82.90)	115.00 (69.30)
No	57.00 (28.40)	6.00 (17.10)	51.00 (30.70)
Dialysate calcium, *n* (%), mmol/L			
Normal (1.5)	111.00 (55.20)	22.00 (62.90)	89.00 (53.60)
Low (1.25)	90.00 (44.80)	13.00 (37.10)	77.00 (46.40)
Serum calcium, mean (SD), mmol/L	2.27 (0.14)	2.30 (0.13)	2.27 (0.14)
Serum phosphate, mean (SD), mmol/L	1.56 (0.42)	1.47 (0.37)	1.57 (0.43)
Serum alkaline phosphatase, mean (SD), U/L	143.73 (159.33)	100.90 (113.91)	152.76 (166.22)
Serum albumin, mean (SD), g/L	39.25 (4.60)	38.91 (5.08)	39.32 (4.50)
Calcium-based phosphate binder use, *n* (%)			
Yes	160.00 (79.6)	30.00 (85.70)	130.00 (78.30)
No	41.00 (20.4)	5.00 (14.30)	36.00 (21.70)
Non-calcium-based phosphate binder use, *n* (%)			
Yes	50.00 (24.9)	1.00 (2.90)	49.00 (29.50)
No	151.00 (75.1)	34.00 (97.10)	117.00 (70.50)
Calcitriol or vitamin D analogue use, *n* (%)			
Yes	119.00 (59.2)	10.00 (28.60)	109.00 (65.70)
No	82.00 (40.8)	25.00 (71.40)	57.00 (34.30)
Calcimimetic use, *n* (%)			
Yes	22.00 (11.00)	0 (0)	22.00 (13.30)
No	179.00 (89.00)	35.00 (100.00)	144.00 (86.70)

Data are *n* (%) for categorical variables, mean [SD] for normally distributed continuous variables and median [IQR] for non-normally distributed continuous variables. Abbreviations: SD, standard deviation; IQR, interquartile range.

**Table 2:** Univariable logistic regression.

Variables	Univariable Model	
	Crude OR (95% CI)	*p* Value *
Age	1.04 (1.01–1.07)	**0.03**
Gender		
Male	0.94 (0.45–1.98)	0.87
Female	Reference	
Etiology of kidney failure		
Diabetes mellitus	Reference	
Hypertension	0.68 (0.24–1.94)	0.47
Chronic glomerulonephritis	0.37 (0.11–1.31)	0.12
Others	1.32 (0.25–6.94)	0.74
Dialysis vintage	1.00 (0.99–1.01)	0.55
Diabetes mellitus		
Yes	2.14 (0.84–5.48)	0.11
No	Reference	
Dialysate calcium, mmol/L		
Normal (1.5)	Reference	
Low (1.25)	0.68 (0.32–1.45)	0.32
Serum calcium, in 0.1 mmol/L	1.21 (0.93–1.57)	0.16
Serum phosphate, mmol/L)	0.54 (0.21–1.37)	0.19
Serum alkaline phosphatase, U/L	0.99 (0.98–1.00)	**0.038**
Serum albumin, g/L	0.98 (0.91–1.06)	0.64
Calcium-based phosphate binder use		
Yes	1.66 (0.60–4.59)	0.34
No	Reference	
Non-calcium-based phosphate binder use		
Yes	0.07 (0.00–0.52)	**0.01**
No	Reference	
Calcitriol or vitamin D analogue use		
Yes	0.21 (0.09–0.47)	**<0.001**
No	Reference	

* Bolded values indicate statistical significance of *p* < 0.05. Abbreviations: OR, odds ratio; CI, confidence interval.

**Table 3:** Multivariable logistic regression.

Variables	Multivariable Model	
	Adjusted OR (95% CI)	*p* Value *
Age	1.02 (0.99–1.06)	0.22
Diabetes mellitus		
Yes	2.32 (0.80–6.69)	0.12
No	Reference	
Dialysate calcium, mmol/L		
Normal (1.5)	Reference	
Low (1.25)	0.55 (0.23–1.33)	0.18
Serum calcium, in 0.1 mmol/L	1.68 (1.15–2.45)	**0.007**
Serum alkaline phosphatase, U/L	1.00 (0.99–1.00)	0.40
Non-calcium-based phosphate binder use		
Yes	0.05 (0.00–0.47)	**0.008**
No	Reference	
Calcitriol or vitamin D analogue use		
Yes	0.20 (0.08–0.50)	**<0.001**
No	Reference	

* Bolded values indicate statistical significance of *p* < 0.05. Abbreviations: OR, odds ratio; CI, confidence interval.

**Table 4:** Comparison of complications between patients with and without adynamic bone disease.

Complications	Adynamic Bone Disease (*n* = 35)	No Adynamic Bone Disease (*n* = 166)	*p* Value
Ischemic heart disease, *n* (%)			
Yes	21.00 (60.00)	77.00 (46.39)	0.143
No	14.00 (40.00)	89.00 (53.61)	
Peripheral vascular disease, *n* (%)			
Yes	5.00 (14.29)	29.00 (17.47)	0.648
No	30.00 (85.71)	137.00 (82.53)	
Stroke, *n* (%)			
Yes	6.00 (17.14)	29.00 (17.47)	0.963
No	29.00 (82.86)	137.00 (82.53)	
Fracture, *n* (%)			
Yes	4.00 (11.43)	30.00 (18.07)	0.341
No	31.00 (88.57)	136.00 (81.93)	
Mortality, *n* (%)			
Yes	3.00 (8.57)	10.00 (6.02)	0.578
No	32.00 (91.43)	156.00 (93.98)	

## Data Availability

The datasets generated and/or analyzed during the current study are not publicly accessible due to privacy and confidentiality considerations. However, anonymized data may be made available by the corresponding author (M.B.) upon reasonable request.
